# NIST Ballistics Toolmark Research Database

**DOI:** 10.6028/jres.125.004

**Published:** 2020-01-20

**Authors:** Xiaoyu Zheng, Johannes Soons, Robert Thompson, Sushama Singh, Cerasela Constantin

**Affiliations:** 1 National Institute of Standards and Technology,Gaithersburg, MD 20899, USA

**Keywords:** 3D topography, database, firearms, toolmark

## 1 Summary

In 2009, a report by the National Academies [1] called into question, amongst other issues, the objectivity of visual toolmark identification by firearms examiners. The National Academies recommended development of objective toolmark identification criteria and error rate estimates. Industry [2,3], academia [4], and government laboratories [5] are pursuing two promising approaches towards this goal: 1) development of mathematical criteria and advanced algorithms for the objective and automated identification and scoring of potential matches, and 2) supplementing traditional reflectance microscopy images with three-dimensional surface topography measurement data.^1^1 Any mention of commercial products within this paper is for information only; it does not imply recommendation or endorsement by NIST.

Development of both these approaches to objective toolmark identification are hindered by a lack of access to toolmark datasets that 1) represent the large variety of ballistic toolmarks encountered by firearm examiners, and 2) represent challenging identification scenarios, such as those posed by consecutively manufactured firearms components. It is not economically feasible for individual companies or institutions to generate their own datasets. This makes it difficult for these entities to objectively evaluate their solutions. The NIST Ballistics Toolmark Research Database (NBTRD) removes this hurdle by creating an open access database where 3D topography and 2D image data of bullet, cartridge case and toolmark surfaces could be shared between researchers, whereby new systems, methods, and algorithms could be tested, refined, and compared. The database also provides the representative variety of toolmark data required, ranging from crime lab test fires to test fires conducted using consecutively manufactured barrels, firing pins, slides and other firearm surfaces. The database contains both reflectance microscopy images and three-dimensional surface topography data along with all its relevant metadata.

The database enables researchers to test new approaches to objective, mathematics-based, toolmark identification while easing the transition to three-dimensional surface topography data. The database will provide a foundation for a scientific knowledge base on the degree of similarity that can be found between marks made by different firearms and the variability in marks made by an individual firearm. The current, ‘fairly limited [1],’ knowledge base is a fundamental barrier to the development and validation of objective mathematical similarity criteria, and associated confidence limits, applicable to a broad range of firearms and ammunition brands.

## 2. Data Specifications

**Table:** 

**NIST Operating Unit(s)**	Physical Measurements Laboratory, Sensor Science Division
**Format**	XML 3D Surface Profile (ISO 25178-72)
**Instrument**	Disc-Scanning Confocal and other 3D Surface Topography Measurement Instruments.
**Spatial or Temporal Elements**	N/A
**Metadata Specifications**	https://tsapps.nist.gov/NRBTD/Content/Documents/NIST-Ballistics-Toolmark-Database-Meta-Data-Glossary-2.pdf
**Accessibility**	Publicly Available
**License**	https://www.nist.gov/director/licensing

## 3 Methods

The NBTRD was designed with four major requirements.

1) Measurement data from a diverse population of test fired bullets and cartridge cases which represents the general population of samples encountered during case work. Also, to include challenging samples from consecutively manufactured firearm components and other designed studies.
2) An in-depth metadata structure to catalogue the measurements based on their class characteristics.
3) An open standard data exchange format for the interoperability of 2D/3D data measured from different measurement systems. This also enables different software packages to utilize a common format.
4) A portal for researchers to upload their own measurement data adding to the expanding knowledge base of firearm toolmarks.

### 3.1 Measurement Data from a Diverse Population of Test Fires

Acquisitions of 3D datasets were conducted at NIST using a disc-scanning confocal microscope. A 20X objective was used for bullet land engraved areas and the cartridge case firing pin impressions. A 10X objective was used for the cartridge case breechface impressions.

Acquisitions of 2D datasets were conducted at NIST using a stereo microscope. For cartridge case firing pin impressions, a 4X objective was used . For the cartridge case breechface impressions, a 2X objective was used . Bullet land engraved areas were not imaged. Measurements conducted by non-NIST institutions have other varying measurement parameters which can be found in the metadata structure of each measurement.

The initial datasets that were uploaded originated from designed firearm toolmark studies that NIST conducted in the past. These included test fires generated from consecutively manufactured pistol slides and barrels, wear studies investigating how the toolmarks on a bullets and cartridge cases change over many firings using the same firearm, and the effect of ammunition types on the impressed toolmarks found on cartridge cases. These datasets served as pilot datasets to help build and improve the database. Additional datasets of similarly designed studies have also been measured and added to the database since its inception.

Outside of data from designed studies, the database includes data from populations of test fires that originated from firearm reference collections. National, state, and local forensic laboratories from around the United States volunteered to test fire their reference collections to help build the NIST Ballistics Toolmark Research Database. The collection targeted the most common handgun calibers 38 Special, .380 ACP, 9 mm Luger, .40 S&W, and .45 ACP. By collecting data from these test fires from around the country, a large, diverse population of data from samples can be collected and used for research targeting objective comparison metrics and statistical weight of evidence development.

### 3.2 Metadata Structure

**Fig. 1:**
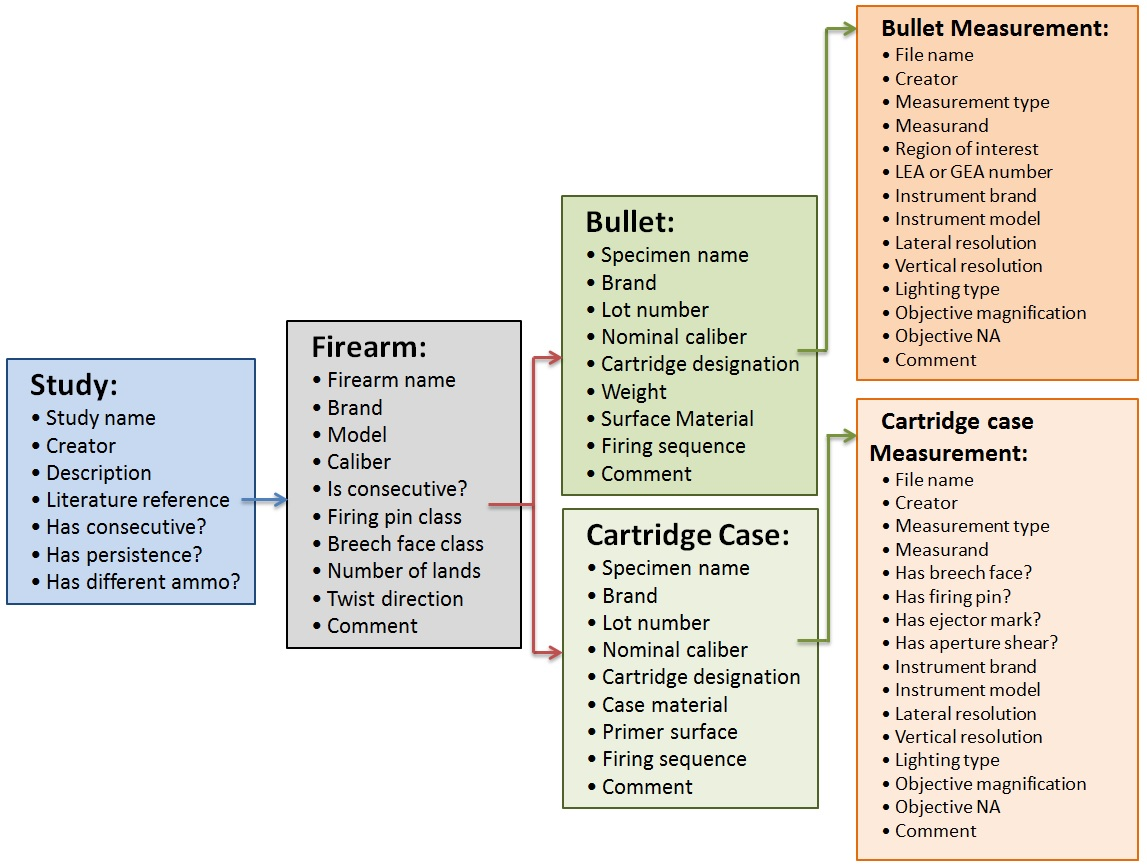
Metadata Hierarchy for NBTRD.

NBTRD is designed as a searchable database. For this to occur, the data must be indexed according to their class characteristics using metadata. Working with forensic firearm examiners, the NBTRD gathered a list of major class characteristics currently being used to catalogue firearm toolmarks and expanded on them. As seen in Figure 1, datasets are organized into “Studies” at the highest level. Metadata at this level describe the creator, references, and other high-level searchable descriptions. Within a “Study”, there must

exist one or more Firearms. Metadata at the “*Firearm*” level describe the brand, model, caliber, and other related class characteristics of each firearm contained within the “*Study*”. Each “*Firearm*” will then have test fired “*Bullets*” and/or “*Cartridge cases*”. Metadata at this level describe the brand, model, weight, surface material and other related class characteristics for each test fire. The last level of the hierarchy contains the “*Measurement*” data. Metadata at this level describe the measurement conditions under which each firearm toolmark was measured. These include the instrument type, measurand, resolutions, and other related parameters.

When a user downloads any data from the database, an attached metadata spreadsheet will be included describing the entire metadata hierarchy traced all the way back to the firearm and study that it came from. Users can also use these metadata fields as a way to search and filter through the entire database returning only the data they are interested in. A full data dictionary and their enumerations can be found at https://tsapps.nist.gov/NRBTD/Content/Documents/NIST-Ballistics-Toolmark-Database-Meta-Data-Glossary-2.pdf

### 3.3 Open Standard Data Exchange Format

All measurement data on the NBTRD are provided as raw data from the acquisition instrument. Depending on the nature of the firearm identification approach, users may have to apply data processing operations such as data trimming, outlier rejection, edge detection, leveling, form removal, and filtering.

2D reflectance microscopy images are provided in lossless greyscale .PNG format.

In an effort to standardize 3D firearm toolmark measurements within the field, NIST along with industry, academic, and forensic researchers formed the Open Forensic Metrology Consortium (OpenFMC). The mission of this consortium is to establish file formats, means of data exchange, and best practices for researchers using 3D metrology in the forensic sciences. This is especially important as more forensic laboratories are starting to adopt these new technologies. The NBTRD and OpenFMC adopted the X3P format (XML 3D Surface Profile) as the standard file exchange format for 3D firearm toolmark measurements. This format is defined in ISO 25178-72 for exchange of surface texture data and has open source read/write functions in C++ and Matlab. The format also enables custom metadata defining measurement parameters and other relevant firearm/ammunition class characteristics. Each X3P file contains the following four standard records:

Record 1: Header, data types, and axes definition
Record 2: Metadata regarding the instrument and user
Record 3: Profile Data (x, y, z)
Record 4: Checksum of the xml-document

### 3.4 User Uploads

The majority of data on the NBTRD was measured and uploaded by NIST. While NIST has access to a lot of test fires and data, to truly sample the firearm populations and instruments out in the field, crowd sourcing additional data is one of the requirements for NBTRD. A secure portal is provided for researchers, practitioners, and instrument manufacturers to have the ability to upload data to the NBTRD in accordance with the metadata structure shown in figure 1. The data must also conform to the standard data exchange formats listed in the previous section. By allowing users to upload data, NBTRD is able to leverage the available firearm toolmark data for researchers all over the world.

## 4 Impact

The research web database infrastructure developed in this project provides a growing, shared, ground truth database on the degree of similarity that can be found between marks made by different firearms and the variability in marks made by an individual firearm. This is achieved through a large variety of challenging datasets representing:

● Test fires conducted using consecutively manufactured barrels, slides, firing pins, and other firearm parts;
● Test fires conducted using the same firearm, with large numbers of intermediate firings to represent varying degrees of firearm wear; and
● Test fires conducted using different brands of ammunitions.

The database contains both test fires characterized using state-of-the-art measurement equipment and measurement protocols at NIST, as well as user-supplied test fires with varying degrees of quality.

The database will spur the development and validation of mathematical criteria, algorithms, and systems for objective firearms identification. This is achieved through a unique focus on challenging scenarios, such as consecutively manufactured firearm components, persistence firings, and different ammunition types. These research datasets cannot be obtained from existing forensic databases such as NIBIN. It is not economically feasible for a single entity, such as a university or system developer, to generate the variety of datasets required for broadly applicable results.

The challenging identification scenarios provide researchers, for the first time, with the large variety of data needed to assess worst-case variability and repeatability, providing a path to the development of scientifically-justified methods that yield identification results with well-characterized, quantitative, confidence limits.

The database entries submitted by NIST are designed to ease the transition to 3D topography data, by providing both image intensity data obtained under various lighting conditions and 3D topography data for the same test fires.

Although not addressed in this project, the database provides additional opportunities for:

1) Quality control of crime lab measurements and support of instrumentation development through supplementary toolmark replicas corresponding to reference images in the database;
2) Government-organized competitions between instrument or software developers with the goal of advancing the technology as quickly as possible, similar to the approach taken for the development of systems for fingerprint identification and for facial recognition [2-4];
3) Validation of correlation software; and
4) Expansion to other types of toolmark data (e.g., screwdriver, chisel, crowbar, or wire cutter).
